# Delivering a family‐based child mental health promotion program among two resettled refugee communities during the COVID‐19 pandemic: Lessons learned in a hybrid type II implementation‐effectiveness randomized controlled trial

**DOI:** 10.1002/ajcp.70021

**Published:** 2025-10-05

**Authors:** Euijin Jung, Candace Black, Matias Placencio‐Castro, Lila Chamlagai, Rilwan Osman, Morgan Hoffman, William Beardslee, Theresa S. Betancourt

**Affiliations:** ^1^ School of Counseling Psychology and Social Welfare Handong Global University Pohang Kyungbuk South Korea; ^2^ Research Program on Children and Adversity Boston College School of Social Work Chestnut Hill Massachusetts USA; ^3^ Behavioral Health and Social Science Department Brown University School of Public Health Providence Rhode Island United States; ^4^ Maine Immigrant and Refugee Services (MEIRS) Lewiston Maine USA; ^5^ Department of Psychiatry Boston Children's Hospital Cambridge Massachusetts USA

**Keywords:** community‐based participatory research, EPIS, family functioning, implementation science, prevention science, Refugees, youth mental health

## Abstract

**Background:**

Resettled refugee families face elevated mental health risks, compounded by structural and cultural barriers. The Family Strengthening Intervention for Resettlement (FSIR), co‐developed with resettled refugee communities, aims to improve family functioning and child mental health. This study evaluated FSI‐R in Somali Bantu and Bhutanese communities in New England during COVID‐19 using a Hybrid Type II Implementation‐Effectiveness Trial guided by the EPIS framework.

**Methods:**

Linear mixed modeling assessed changes in family functioning and child mental health. A process evaluation identified implementation barriers and informed adaptations. Activities were registered under Clinical Registry #NCT03796065.

**Results:**

Bhutanese families receiving FSI‐R showed greater improvements in parental supervision compared to usual care. Process evaluation highlighted that responsiveness to community needs supported successful implementation despite pandemic stressors. Somali Bantu interventionists reported stronger emotional connection with families during in‐person delivery.

**Conclusions:**

Findings support the utility of hybrid trials in assessing both effectiveness and implementation of preventive interventions with resettling families. Despite contextual disruptions, attention to community needs and delivery flexibility enabled successful implementation. This study underscores the importance of context‐informed strategies to sustain core elements of evidence‐based interventions in dynamic settings.

## Key Highlights

- Community‐based participatory research helped overcome barriers through collaborative, context‐informed implementation guided by the EPIS implementation science framework.
- FSI‐R improved parental supervision in Bhutanese families during a period of high stress due to the global COVID‐19 pandemic.
- The Hybrid Type II implementation‐effectiveness design supported real‐time adaptation to policy shifts and community needs.

**Abbreviations**
APQ: alabama parenting questionnaire
CAU: care as usual
CBPR: community‐based participatory research
CES‐DC: center for epidemiologic studies depression scale for children
FSI‐R: family strengthening intervention for refugees
WHODAS: World Health Organization Disability Assessment Schedule

## INTRODUCTION

Refugee children and families resettling in the United States are at elevated risk of developing mental health problems and face structural and cultural barriers to accessing care (Abdi et al., 2022). These realities underscore the need for evidence‐based interventions to promote refugee mental health (DiClemente‐Bosco et al., 2023; Ellis et al., 2014). Improving family support and functioning in raising school‐aged children can make a major contribution to this goal, especially when delivered by peers with similar cultural and linguistic backgrounds as well as shared lived experience of resettlement.

Studies of refugee children and adolescents have documented much higher levels of depression, anxiety, and posttraumatic stress disorder (PTSD) compared to the nonrefugee populations (Bogic et al., 2015; Henkelmann et al., 2020; Kien et al., 2019; Mesa‐Vieira et al., 2022). The elevated risk of developing mental health problems may be attributable to traumatic experiences such as exposure to armed conflict and the loss of family members in the premigration and displacement phases of the refugee journey (Lustig et al., 2004), as well as acculturative and contextual stress associated with adjusting to new lives in resettlement (Betancourt et al., 2015; Bogic et al., 2015; Jorgenson & Nilsson, 2021). Challenges in the resettlement context often include very real struggles for caregivers to find adequate work and housing as well as adjusting to a new language and culture, which may pose difficulties for also attending to the adjustment and mental health needs of their children.

In the United States in recent years, rising nationalism, racism, and xenophobia has targeted refugee and immigrants when combined with the global COVID‐19 pandemic, risk factors facing these populations have all been accentuated (Dubey et al., 2020; Hynie, 2018; Júnior et al., 2020; Liu et al., 2013; McGuire et al., 2021). To help counteract the many risk processes shaping refugee child and adolescent mental health, the need to promote healthy family functioning and strong parent–child relationships among resettled families can present a major opportunity for mental health promotion (Betancourt et al., 2020; Bunn et al., 2022; Ellis et al., 2014; Taylor et al., 2014). Research on effective evidenced‐based interventions for improving family functioning and child mental health in resettled communities suggests that interventions led by refugee peers with lived experience can help to address important gaps in existing mental health services that often lack culturally‐ and linguistically adapted intervention models among the diverse cultural groups resettling in the United States. Given this background, the Family Strengthening Intervention for Resettlement (FSI‐R) was adapted from the evidence‐based Family Talk intervention for family‐based promotion of mental health in children across diverse cultures and settings (Furlong et al., 2024; Lövgren et al., 2022; Pihkala et al., 2012). In the present study, FSI‐R was co‐developed using a Community‐Based Participatory Research (CBPR) approach and tested as a peer‐delivered prevention model for children (aged 7–17) and their caregivers from two distinct resettling populations.

### Somali Bantu and Bhutanese refugees

This project arose from long‐term partnerships with resettled Somali Bantu and Bhutanese refugee communities in New England. The Somali Bantu are ethnic minorities who were trafficked and enslaved in other parts of Africa during the nineteenth century (Lehman & Eno, 2003). When the Somali civil war erupted in 1991, the Somali Bantu were forcibly displaced from their homes to refugee camps in Kenya. The Somali Bantu have a long history of persecution, historically limited access to education, and have inhabited some of the most vulnerable parts of two major Kenyan refugee camps (Daadab and Kakuma) for generations (Lehman & Eno, 2003; Menkhaus, 2003).

The Lhotshampa Bhutanese are an ethnic Nepalese minority who lived for generations in southern Bhutan. In September 1990, the Kingdom of Bhutan expelled the Lhotshampa population under “Bhutanization,” or ethnic cleansing campaigns involving acts of violence and loss of property rights (Hutt, 1996). The evicted Lhotshampas settled in refugee camps in the eastern part of Nepal, where many have remained for decades (International Organization for Migration, 2008). In 2008, large‐scale resettlement of Lhotshampa Bhutanese refugees began in the United States. The mental health needs of this group have been underscored by the high rates of suicide, which at its peak was nearly twice that of the US national average (24.4 per 100,000) (Cochran et al., 2013).

### CBPR and the EPIS framework for implementation science of evidence‐based interventions

The EPIS (Exploration, Preparation, Implementation, and Sustainment) framework (Aarons et al., 2011) guided the present Hybrid Type II Implementation‐Effectiveness Trial. Hybrid designs^1^ simultaneously assess effectiveness and implementation strategies, providing critical information to support program scaling and sustainment (Curran et al., 2012). The hybrid design enabled us to assess not only whether the FSI‐R improved family functioning and child mental health within two unique refugee communities, but also how well the program could be implemented in these different community contexts during a highly challenging period characterized in part by the COVID‐19 pandemic and restrictive refugee policies. EPIS is an implementation science framework that facilitates assessment of implementation barriers and facilitators in the inner and outer contexts of the delivery ecosystem involved in extending the reach and sustaining quality in the delivery of evidence‐based mental health interventions (EBIs). Within this framework, major bridging factors that link the outer political and cultural context with inner features of the intervention, agencies, and communities involved in the EBI implementation included a stance grounded in CBPR. EPIS has been used in a variety of public sectors, including mental health, education, and child welfare (Moullin et al., 2019), as well as in youth and family‐based interventions in culturally diverse and underserved settings (Black et al., 2025; Bond et al., 2022; Desrosiers et al., 2024; Freeman et al., 2024), because of its emphasis on contextual fit and sustainability. EPIS is well suited for interventions like FSI‐R that require adaptation to unique cultural, linguistic, and community needs, such as those of the Somali Bantu and Bhutanese refugee families involved in this study.

This Hybrid Type II trial emerged from a multi‐year CBPR approach which co‐adapted the EBI with refugee individuals with lived experience and built connections between partner organizations, families, and other health and social services offered within these agencies. This project was co‐led by the Boston College School of Social Work Research Program on Children and Adversity and two long‐standing refugee community‐based organizations (CBOs). These two CBOs played central roles in recruiting, staff hiring and training, and fostering collaboration between local community members and university researchers.

**Figure 1:**
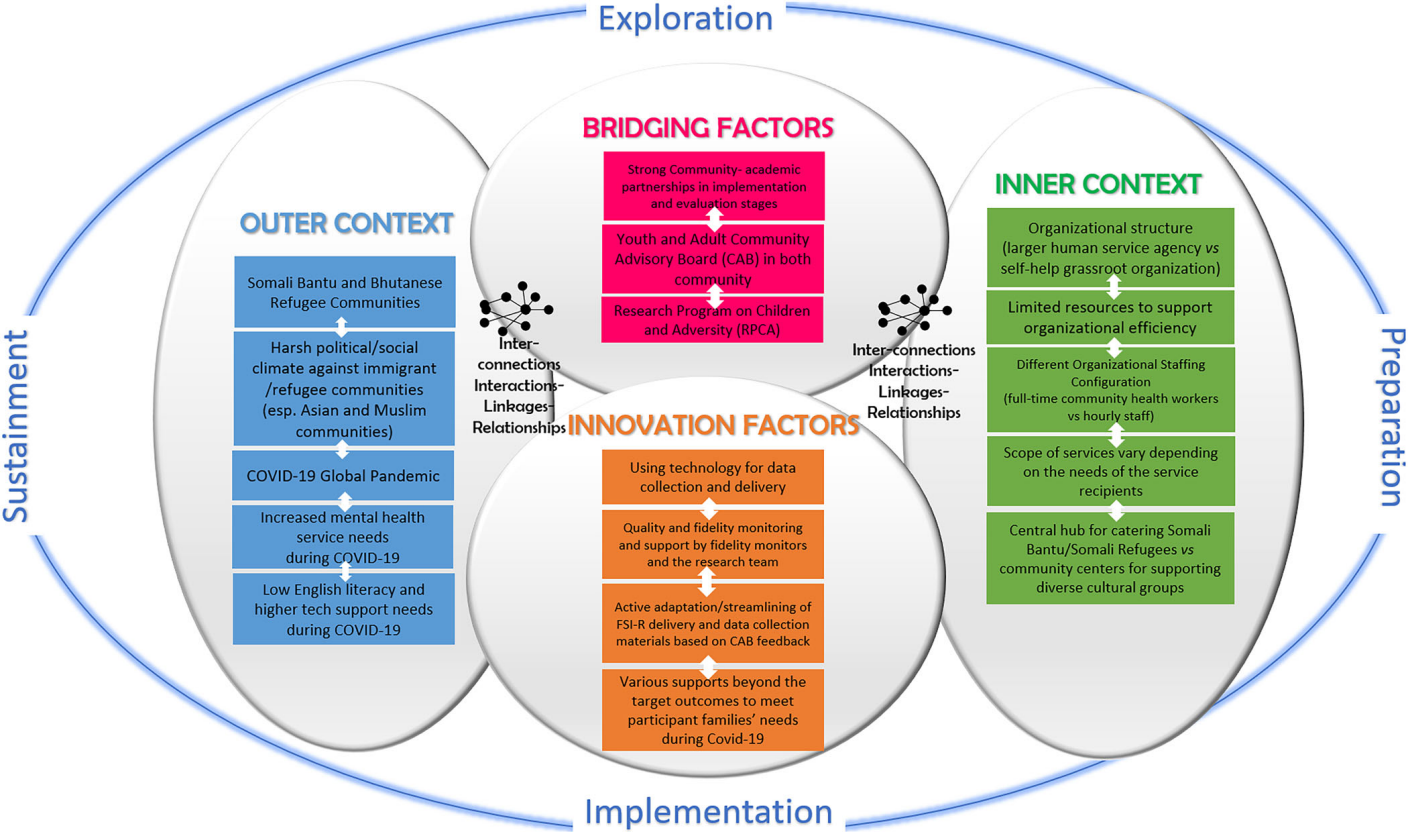
Using EPIS framework for FSI‐R Implementation. *Adapted from Moullin, J. C., Dickson, K. S., Stadnick, N. A., Rabin, B., & Aarons, G. A. (2019). Systematic review of the Exploration, Preparation, Implementation, Sustainment (EPIS) framework. Implementation Science, 14(1), 1.

As mapped under the EPIS implementation model (Figure 1), each of our CBOs had distinct organizations exhibiting contrasting organizational structure, characteristics, and different staffing configurations. The first organization was linked to a local branch of a large human services agency affiliated with a national network working on refugee resettlement, which had been founded with the purpose of catering to a wide range of resettlement needs from many cultures. The agency had developed and implemented a range of public health initiatives serving refugee and migrant communities and had greater and sophisticated administrative and policy operations. The other CBO was a grassroots organization, founded by local refugee community members, that serves as a central hub for providing assistance to resettling populations, mainly Somali Bantu and Somali.

Community advisory board (CAB) meetings were an important mechanism for reviewing implementation barriers and arriving at culturally resonant proposed solutions to implementation barriers at each of the two implementation sites which were then analyzed using Plan‐Do‐Study‐Act (PDSA) cycles. CAB meetings using PSDA cycles suggested strategies for overcoming barriers which were then implemented and examined for impact. Successful strategies for overcoming implementation barriers were then shared across both sites. The two CBOs demonstrated flexibility in adapting the program to effectively address the needs of each of the resettling communities. Unfortunately, due to logistics of working across two states, we were unable to vary cultural and language group across sites so the pre‐existing CHWs served Bhutanese refugee families while the peers hired temporarily for the services research implemented in the Somali Bantu community.

As the present study was conducted during a time of unexpected and unprecedented political and societal upheaval, EPIS was used to critically review how internal and external factors affected program implementation, barriers and facilitators to successful implementation while also looking at effectiveness outcomes using a randomized design embedded within a CBPR approach.

#### Outer context

This study was conducted between February 2019 and June 2022, years of unprecedented upheaval affecting the lives of immigrant and refugee communities further disrupted by the emergence of the COVID‐19 Pandemic. During the time of the study implementation, the first Trump administration introduced a bill that that would cut legal immigration by 50% and cap refugee admissions at 45,000 per year (Martin, 2018). The refugee admissions were cut significantly from 84,994 in Fiscal Year (FY) 2016 to 11,411 in FY 2021 (Refugee Processing Center, 2024).

In a period of increasingly restrictive immigration policy, entry was suspended for individuals from several Muslim‐majority countries, including Somalia, along with a temporary halt to all refugee admissions. These restrictions were later expanded, resulting in a significant drop in admissions from 11 countries—9 of which were majority Muslim—due to added application barriers and a pause on a family reunification program (De Peña & Corte, 2019; Kerwin & Nicholson, 2021).

Furthermore, the advent of the COVID‐19 pandemic was particularly harmful for the mental health of refugee families (Dubey et al., 2020; Júnior et al., 2020; McGuire et al., 2021). While the COVID‐19 pandemic contributed to poor population mental health overall—including a threefold increase in the prevalence of depression (Ettman et al., 2020)—public lockdown measures and social isolation were found to trigger distressing traumatic memories for many refugees who had experienced previous stay at home orders sue to insecurity as well as forced relocation (Brickhill‐Atkinson & Hauck, 2021; Browne et al., 2021; Kiteki et al., 2022). During the pandemic, refugee families reported a heightened sense of loneliness and isolation (Browne et al., 2021).

#### Innovation factors

**Figure 2:**
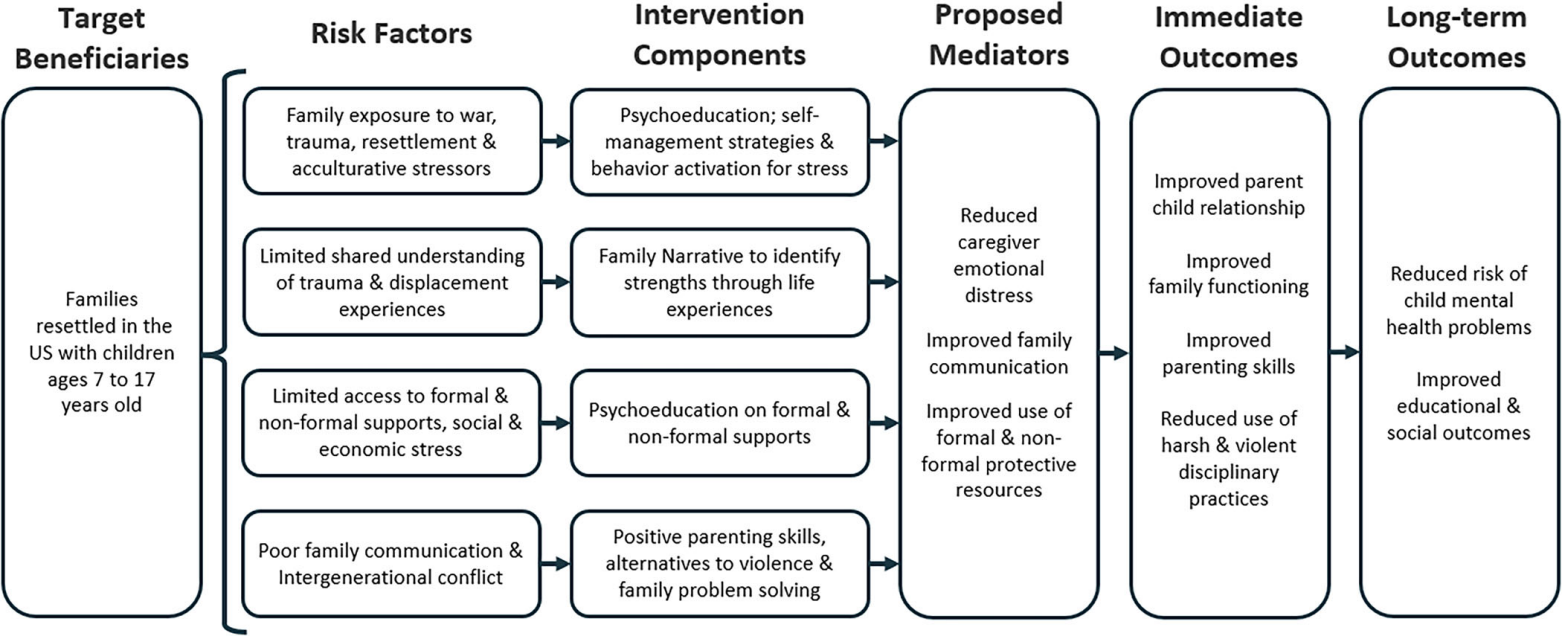
Theory of change model for FSI‐R. FSI‐R, Family Strengthening Intervention for Resettlement.

The FSI‐R was adapted from the Family‐Based Preventive Intervention/Family Talk (Beardslee et al., 1996), originally developed to mitigate the risk of child depression in the offspring of depressed caregivers. The intervention had also demonstrated success in reducing depression among children and adolescents exposed to adversity in sub‐Saharan Africa (Betancourt et al., 2017). Beginning in 2004, we used CBPR methods to engage resettled refugee community members in needs assessments that shaped intervention co‐adaptation. In collaboration with Somali Bantu and Bhutanese refugees, we developed 10 culturally tailored modules to improve family functioning including parent–child communication, monitoring, navigation of school and community relationships, reduce intergenerational conflict and, per theory, promote mental health in children aged 7–17 (Figure 2).

The FSI‐R modules comprise elements such as a family narrative that emphasizes strengths that have helped the family navigate difficult experiences in the past and a family meeting facilitated by caregivers at the end of the intervention modules during which families review their strengths and challenges as a family, as well as their future aspirations and goals (DiClemente et al., 2023; see Supporting Information S1: Appendix I).

The FSI‐R was designed to support a task‐sharing model (Hoeft et al., 2018; Mendenhall et al., 2014) wherein the intervention could be delivered by individuals with diverse backgrounds, including nonspecialist peers with lived experience and cultural and linguistic similarity to the populations being served. Each module is delivered during home visits in the participants' preferred language (Nepali, Maay Maay, Somali, and English). Quality of delivery was ensured via ongoing training and monitoring of the nonspecialist peer home visitors including weekly on‐site supervision as well as cross‐site exchange and case presentations during group “super‐supervision.”

A prior randomized pilot of the FSI‐R with Somali Bantu and Bhutanese communities (Betancourt et al., 2020) indicated that children who received the FSI‐R intervention had significantly reduced traumatic stress reactions (*β* = −0.42, *p* = .03), while caregivers reported less depression symptoms in their children (*β* = −0.34, *p* = .001) compared to Care‐As‐Usual families. In addition, Bhutanese children receiving FSI‐R reported lower family arguing (*β* = −1.32, *p* = .04) while Bhutanese caregivers reported lower conduct problems in their children (*β* = −9.20, *p* = .01).

### Current study

In this study, we report findings from a Hybrid Type II Implementation‐Effectiveness Study (Clinical Registry #NCT03796065). A randomized control trial (RCT) simultaneously tested effectiveness and implementation strategies of the FSI‐R in improving family functioning and mental health outcomes in Somali Bantu and Bhutanese resettled refugee children and adolescents. Primary outcomes included family communication, intergenerational conflict, and parenting behaviors, and secondary outcomes are internalizing and externalizing problems in youth aged 7–17. Implementation outcomes included fidelity, competence scores, and outcomes on the System for Observing Family Therapy Alliances (SOFTA) scale.

## METHODS

### Staff recruitment, training, and supervision

Staff were recruited by both CBOs based on their cultural affinity, language skills, and shared lived experiences with families served. Two types of staffing structures were examined: one involved nonspecialist home visitors, recruited and trained specifically for this project and the other involved training extant nonspecialist community health workers. Eight home‐visitors (“interventionists”), four from each CBOs, received intensive, in‐person, role‐play‐based training (80 h) before program delivery. Research assistants (RAs), also from the same communities, were trained in ethics and research procedures. RAs were responsible for collecting data at baseline, immediately post‐intervention (midline), and 6‐month follow‐up. REDCap software (version 10.3.4; Harris et al., 2019) on Android device was used for data collection, case management, tracking of appointments, referrals, and contact information.

Ongoing, weekly clinical supervision was conducted by the research team and clinical supervisor at each organization. On‐site clinical supervisors had Master's‐level social work degrees and extensive clinical experience with refugees. Supervision sessions focused on overcoming logistical or implementation‐related challenges. Home‐visits were audio‐recorded for review for quality assessment by two expert fidelity monitors with previous experience delivering FSI‐R. Interventionist delivery was rated using a standardized evaluation tool that included items on fidelity (i.e., adherence to the intervention design) and competence (i.e., general home‐visiting session facilitation skills). This feedback was discussed with members of the research team, before providing direct feedback to interventionists during one‐on‐one meetings. Coordination between the research team and fidelity monitors ensured feedback promoted collaborative learning and quality improvement.

### CABs

Following CBPR principles, CABs (both youth and adult groups) were established at each site to incorporate cultural insights and mutually communicate a community's concerns together, providing feedback about implementation contexts (e.g., community factors such as stigma against mental health, gender roles, and barriers to male engagement). The CABs served as a crucial means of problem‐solving and gaining insights to enhance strategies for program delivery (e.g., incorporating simpler local language in manuals, more verbatim instructions, or tips on what should be communicated). The meetings were held quarterly or more frequently as needed (such as during the COVID‐19 outbreak where more than one consultation per month was arranged to implement community psychoeducation on health promotion).

### Family recruitment

The recruitment process began with word‐of‐mouth referrals from CAB members. The CABs helped identify families, enabling enrollment. Organizational leadership also utilized personal networks for referrals and recruitment. Then, RAs reached out to families to invite them to join the FSI‐R study via informed consent. Inclusion criteria were: (1) having formal US government refugee or asylum status; (2) having at least one school‐aged child (7–17 years); and (3) residing in the United States for at least 3 months. Families were excluded if they were found to be experiencing severe crisis at the time of enrollment (e.g., active suicidality or psychosis, divorce proceedings) and were instead referred to a higher level of services.

### Impact of COVID‐19 on FSI‐R implementation

Study enrollment began in February 2019 with projected completion in June 2022. In March 2020, the unmitigated spread of COVID‐19 was underway, leading to quarantine orders in states of implementation. Massachusetts and Maine declared states of emergency order on March 10 and 17. On March 20, the university where the research team was based halted face‐to‐face interactions in human subjects research, discontinuing home‐visits. Study enrollment paused while staff worked to develop and seek approval for protocols for remote enrollment, delivery, and data collection. Several implementation barriers arose, ranging from adapting consent processes, to overcoming poor family engagement during remote program delivery and data collection, and developing a system to ensure incentives could be provided remotely. We provided both interventionists and participants with tablets for virtual sessions. When participants requested in‐person meetings, these were conducted following public health protocols.

Also, to ensure the quality of services, we continued providing super‐supervision sessions that applied PDSA cycles to study barriers and come up with solutions. The team tested and shared back results of solutions as implemented weekly. Successful strategies were shared across sites.

To mitigate survey fatigue with remote data collection during the height of the pandemic, the team pursued CAB and IRB approval to reduce the number of respondents to a single caregiver and a single child per household. To assist RAs with limited technological experience in delivering assessments and family sessions remotely using video conference software, we developed guidance on utilizing tablets and gathering data on sensitive subjects.

Nonetheless, ongoing stay‐at‐home orders contributed to mental health stressors in participating families, with many refugee parents unfamiliar with remote learning or the use of laptops to connect to schools. Even though free laptops were distributed to all school‐aged children enrolled in the public schools in both states, child protection complaints were levied at some refugee parents who were accused of not providing adequate facilitation for their children to join online classroom sessions. Also, many refugee parents reported feeling triggered and experiencing posttraumatic stress reactions to past trauma as well as fear and distrust aggravated by growing anti‐immigrant sentiments and anti‐immigrant/refugee rhetoric common in social media and public discourse at the time. These factors slowed implementation and data collection considerably; ultimately the pace did not recover to pre‐pandemic levels.

### Ethical information

The Boston College Institutional Review Board (Protocol #18.251.01) granted permission for the study and all subsequent modifications to respond to stressors due to the global COVID‐19 Pandemic. Informed consent was obtained from all participants. Oral consent was obtained from adults and signed by RAs as witnesses; for youth participants, parental oral consent and youth oral assent were obtained. Participants received a financial incentive ($50 for adults and $20 for children) for their participation in data collection at all time points.

### Participants

**Figure 3:**
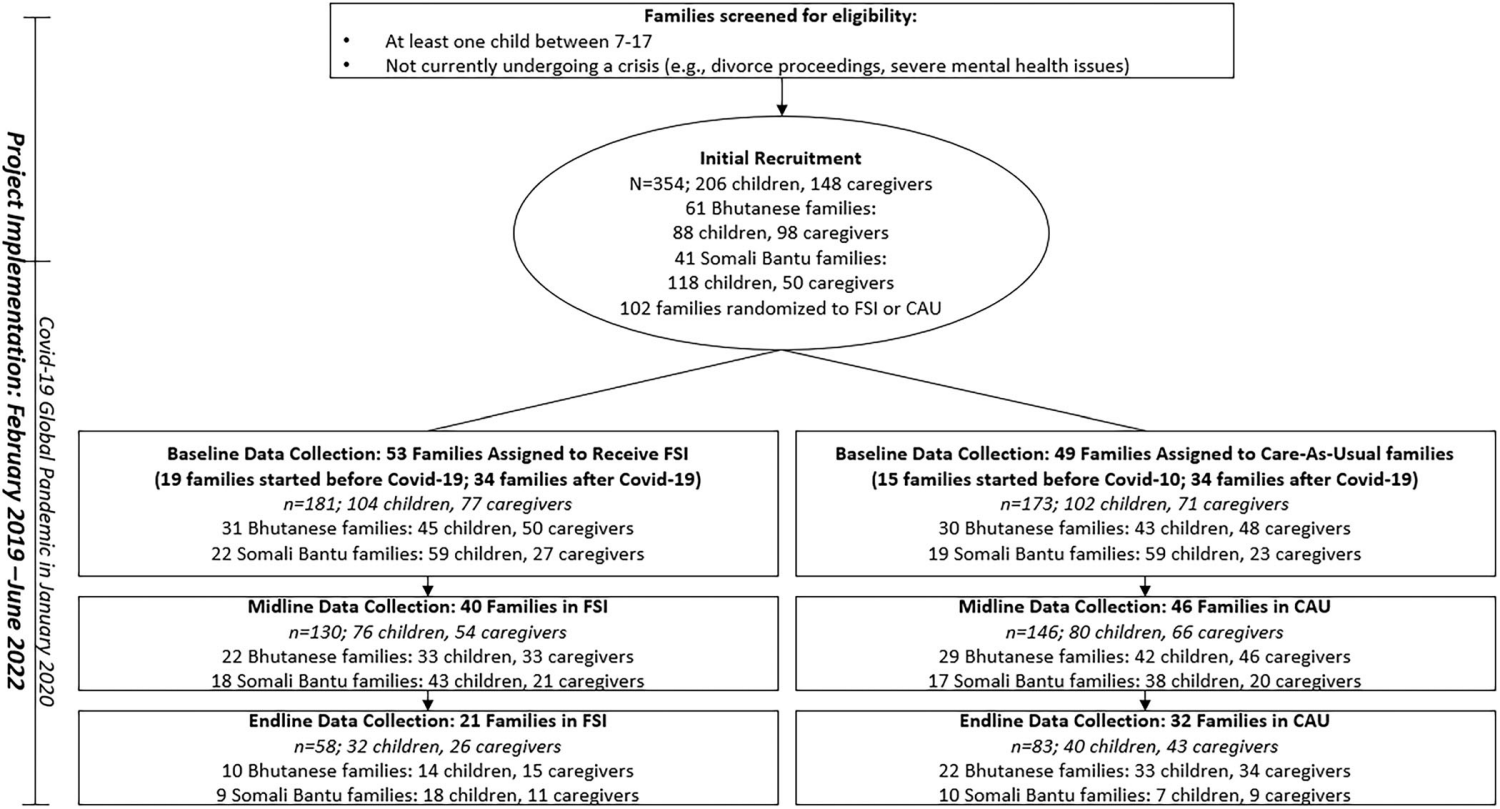
CONSORT flow diagram.

Target enrollment was initially 300 families (150 families from each community) but had to be scaled down to the final sample of 102 families at baseline data collection (354 individuals: 50 male and 98 female caregivers; 102 male and 104 female children). Due to the aforementioned challenges in recruitment and retainment, follow‐up (the third timepoint) sample size was 53 families (141 individuals: 22 male and 47 female caregivers; 37 male and 35 female children) (Figure 3).

Randomization to FSI‐R intervention or Care‐As‐Usual (CAU) was completed using a randomization procedure and data collection via REDCap on tablets with family enrollment in the intervention proceeding as soon as possible following baseline data collection. CAU families were eligible for all routine care services, and intervention families received the FSI‐R program in addition to routine care.

Of enrolled families, 59.8% were from the Bhutanese community, and 40.2% were from the Somali Bantu community. While a third of families started receiving the FSI‐R before the onset of the COVID‐19 Pandemic, the majority of participants received the FSI‐R during the pandemic, including 64% of Bhutanese families and 78% of Somali Bantu families. Despite the pre‐specified plan to collect post‐intervention and 6‐month follow‐up data after intervention completion, these goals were hampered by the COVID‐19 Pandemic and the team was not able to fully collect all data points, especially the 6‐month follow up data. In the end, 40 FSI‐R families (71.8%) and 46 CAU families (84.4%) completed midline data collection post intervention, and 21 FSI‐R families (32.0%) and 32 CAU families (48.0%) completed the 6‐month follow‐up data collection (Figure 3).

### Materials

Assessment batteries comprised tools refined during the pilot study (Betancourt et al., 2020) and both forward‐ and backward‐translated in Maay Maay, Somali, and Nepali, following WHO translation guidelines (Betancourt et al., 2014; WHO, n.d).

#### Effectiveness outcomes

Family conflict was measured using a four‐item family conflict scale (Fosco et al., 2016). Items included “We got angry at each other;” or “We argued” (caregiver report on child: *Cronbah's alpha (here after α*) = 0.74; child report: *α* = 0.83). Youth report of parent‐child communication was assessed with the revised Parent‐Adolescent Communication Form from the Pittsburgh Youth Study (Loeber et al., 1998; *α* = 0.83). Items included “How often does caregiver listen to you?” and “How often does caregiver try to understand your point of view?” Family functioning was measured using the Intergenerational Congruence Measure (Ying et al., 2004) and an adapted version of the Alabama Parenting Index (APQ; Essau et al., 2006). Intergenerational congruence items included “Caregiver and I agree on types of friends I have” or “Caregiver and I generally talk things over together” (caregiver report on child: *α* = 0.78–0.88; child: *α* = 0.88). APQ measures parental involvement, positive parenting, parental monitoring, inconsistent discipline, and corporal punishment practices (caregiver report on child: *α* = 0.87–0.90; child: *α* = 0.85).

Youth externalizing behaviors were measured by an adapted version of the War Affected Youth Psychosocial Assessment (WAYPA; Frounfelker et al., 2025 *;* Betancourt et al., 2014). Items include such as “I fight,” “I use bad language,” or “I misbehave.” The WAYPA was reported by both caregivers and children (caregiver report on child: *α* = 0.11; child: *α* = 0.82). Youth depression was measured using the 20‐item Center for Epidemiologic Studies Depression Scale for Children (*CES‐DC*) (Radloff, 1977) (caregiver report on child: *α* = 0.85; child: *α* = 0.93).

#### Implementation outcomes

Implementation outcomes included quality of delivery items (fidelity and competence) and quality of client‐service provider relationships using the System for Observing Family Therapy Alliances (SOFTA) scale (*α* = 0.88). Fidelity and competence measures were adapted from the original Family Talk intervention (Beardslee et al., 1996). Fidelity and competence were assessed by Seed Team expert fidelity monitors who reviewed audio recordings of sessions conducted in the local language uploaded by home visitors to a secure server. Fidelity items comprised questions specific to each module learning objectives, such as “understanding and defining resilience,” “discussed family challenges and strengths from children's perspectives,” or “discussed family coping with resettlement challenges” (*α* = 0.72–0.95). Competence was measured by the same five items across the module that measured soft skills that support family engagement during intervention (*α *= 0.76–0.97). Competence items included “completed opening and closing clearly,” “encouraged participation from all individuals,” or “used respectful and positive communication.” The SOFTA is an interventionist‐reported measure that aims to understand interventionist–participant collaborative relationship on the intervention program (Cervantes Camacho et al., 2020). SOFTA has 16 items with four subdimensions (engagement in the process, emotional connection, safety, and shared sense of purpose).

### Statistical analysis

Effectiveness of FSI‐R was evaluated using longitudinal linear and logistic mixed‐effects models (Singer & Willett, 2003), for continuous and binary outcomes, respectively. Two‐level models were specified with measurement waves (e.g., timepoints) nested within subjects. Covariates included child's biological age at baseline (continuous), participant gender (1 = female, 0 = male), the number of session attendance (continuous), the proportion of sessions completed remotely (%), and self‐reported stress‐level change after the COVID‐19 pandemic.

The coefficient of interest was the cross‐level interaction between timepoint (baseline, postintervention, 6‐month follow‐up) and intervention status (FSI‐R, CAU), which represents the “difference‐in‐difference” (DID), or the difference in slopes between treatment groups from baseline to post‐intervention or baseline to follow‐up.

Data were analyzed under intention‐to‐treat (ITT) assumptions, meaning that once a family was randomized to CAU or FSI‐R, they were analyzed as belonging to that group despite level of exposure to the intervention. To address attrition, we conducted multiple imputation using chained equations (Plumpton et al., 2016). The majority of data were collected during the COVID‐19 pandemic; thus, our analyses followed best practices from the field to account for the COVID‐19‐induced missingness (Meyer et al., 2020) by the pandemic‐related events and adaptations that had to be made in light of the pandemic. Implementation outcomes were analyzed using descriptive summary statistics. All analyses were done in Stata 18.0 (StataCorp, 2023).

## RESULTS

### Baseline characteristics

Sample characteristics at baseline are displayed in Table 1. Among adult caregivers, 59.18% were female in Bhutanese families and 80% in Somali Bantu families. The average age of caregivers was similar—around 40 years old in both communities. Family size was smaller in Bhutanese families (mean number of children = 2.15) compared to Somali Bantu families (mean number of children = 5.36). On average, Somali Bantu families had been in the United States longer than Bhutanese families (Bhutanese child mean = 5.03 years (SD = 2.67), caregiver = 5.24 years (SD = 2.62); Somali Bantu child mean = 10.77 years (SD = 2.80) adult = 13.76 (SD = 3.06)), and the proportion of family members with US citizenship was much higher among Somali Bantu families than Bhutanese families (Bhutanese child = 17.77%, caregiver = 10.20%; Somali Bantu child = 94.83%, caregiver = 90.0%).

**Table 1:** Baseline characteristics by community.

	Bhutanese (61 families/186 participants)		Somali Bantu (41 families/168 participants)	
	Children	Caregivers	Children	Caregivers
Individuals	88	98	118	50
Female, *n* (%)	42 (47.73)	58 (59.18)	62 (52.54)	40 (80.0)
Age, mean (SD)	12.02 (3.19)	39.52 (8.68)	11.91 (2.87)	42.28 (9.74)
Birthplace countries, *n* (%)				
Bhutan	6 (6.82)	85 (86.73)	.	.
Nepal	72 (81.82)	9 (9.18)	.	.
Somalia	.	.	2 (1.69)	48 (96.0)
Kenya	.	.	18 (15.25)	1 (2.0)
US	5 (5.68)	1 (1.02)	98 (83.05)	1 (2.0)
Other	5 (5.68)	3 (3.06)	.	.
Number of siblings, mean (SD)	2.22 (0.67)		6.08 (2.14)	
Number of children, mean (SD)		2.15 (0.69)		5.36 (2.40)
Years in the United States, mean (SD)	5.03 (2.67)	5.24 (2.62)	10.77 (2.80)	13.76 (3.06)
US Citizenship, *n* (%)	13 (17.77)	10 (10.20)	110 (94.83)	45 (90.0)

Analysis of outcomes at baseline revealed differences between refugee communities (Table 2). Results were collected from 206 participants in total. Overall, Somali Bantu families showed higher scores on dimensions such as family communication, intergenerational congruence, positive parenting, parental monitoring than Bhutanese, while Bhutanese children reported lower depression and externalizing behavior (Table 2). More details on outcomes of interests at each timepoint by intervention status are provided in Supporting Information S1: Appendix II.

**Table 2:** Baseline child and caregiver outcomes by community at baseline.

Outcomes	Bhutanese (*n* = 88)	Somali Bantu (*n* = 118)	*t*(*df*)	*p*‐value
	Mean (SD)	Mean (SD)		
*Family outcome at baseline*				
Family conflict (Primary Outcome 1)				
Child self‐report	0.19 (0.58)	1.17 (1.43)	−5.14 (140)	<.001
Report on child	0.13 (0.31)	0.40 (0.64)	−3.43 (141)	<.001
Communication (Primary Outcome 2)				
Child self‐report	3.35 (0.65)	3.87 (0.84)	−4.00 (137)	<.001
Caregiver report on child	3.09 (1.01)	3.55 (1.21)	−2.43 (140)	0.02
Intergenerational congruence (Primary Outcome 3)				
Child self‐report	4.84 (8.21)	8.36 (12.33)	−1.96 (142)	0.052
Caregiver report on child	3.05 (0.87)	3.74 (0.84)	−5.53 (191)	<.001
Parental Involvement (Primary Outcome 4.1)				
Child self‐report	2.98 (0.67)	3.29 (1.11)	−1.95 (134)	0.053
Caregiver Report on child	3.44 (0.52)	3.62 (0.81)	−1.73 (193)	0.08
Positive Parenting (Primary Outcome 4.2)				
Child self‐report	3.17 (0.76)	3.93 (1.00)	−4.96 (135)	<.001
Caregiver report on child	3.61 (0.63)	4.60 (0.45)	−12.37 (181)	<.001
Lower Parental Monitoring (Primary Outcome 4.3)				
Child self‐report	1.82 (0.59)	1.84 (0.70)	−0.1 (135)	0.83
Caregiver report on child	1.78 (0.66)	1.48 (0.56)	3.44 (196)	<.001
Inconsistent Discipline (Primary Outcome 4.4)				
Child self‐report	1.65 (0.64)	1.98 (0.58)	−3.16 (130)	<.01
Caregiver report on child	1.67 (0.66)	1.89 (0.49)	−2.77 (195)	<.01
Corporal Punishment (Primary Outcome 4.5)				
Child self‐report	1.23 (0.43)	1.22 (0.51)	0.07 (141)	0.94
Caregiver report on child	1.19 (0.48)	1.06 (0.20)	2.46 (197)	0.01
*Child outcome at baseline*				
Externalizing Behavior (Secondary Outcome 1)				
Child self‐report	1.15 (0.19)	1.38 (0.3)	−5.13 (121)	<.001
Caregiver Report on child	1.07 (0.14)	1.23 (0.26)	−4.53 (141)	<.001
Depression (CES‐DC) (Secondary Outcome 2)				
Child self‐report	0.15 (0.18)	0.37 (0.56)	−3.08 (132)	<.01
Caregiver Report on child	0.08 (0.12)	0.11 (0.23)	−0.73 (138)	0.47

### Mixed models

The estimated coefficients and confidence intervals of the mixed models by communities are presented in Table 3. Notably, poor parental monitoring was significantly reduced after the FSI‐R intervention at midline data collection among Bhutanese FSI‐R caregivers compared to CAU group (*B* = −0.49, 95% CI = [−0.96, −0.02]). Among Bhutanese FSI‐R caregivers, the level of child and adolescent monitoring improved compared to CAU, but the same pattern was not observed among Somali Bantu families. No other results were found to indicate a statistically significant improvement, although many results (family conflict, family communication, parental involvement among Bhutanese, positive parenting, child depression) were observed to trend in the hypothesized direction, consistent with the Theory of Change model (Figure 2).

**Table 3:** Estimated difference‐in‐differences coefficients for continuous and binary outcomes.

Outcomes	Bhutanese		Somali Bantu	
	Baseline to Midline [95% CI]	Baseline to Endline [95% CI]	Baseline to Midline [95% CI]	Baseline to Endline [95% CI]
*Family outcome*				
Family conflict (Primary Outcome 1)				
Child self‐report	−0.09 [−0.58, 0.39]	−0.02 [−0.52, 0.48]	0.30 [−0.65, 1.26]	0.15 [−0.81, 1.10]
Report on child	−0.00 [−0.41, 0.40]	−0.03 [−0.58, 0.52]	−0.24 [−0.79, 0.31]	−0.13 [−1.00, 0.74]
Parental efforts for communication (Primary Outcome 2.1)				
Child self‐report	0.03 [−0.47, 0.52]	0.02 [−0.48, 0.52]	0.12 [−0.37, 0.61]	0.05 [−0.53, 0.62]
Parent‐Child Open Communication (Primary Outcome 2.2)				
Child self‐report	−0.45 [−1.21, 0.31]	−0.41 [−1.22, 0.40]	−0.07 [−0.98, 0.84]	−0.41 [−1.40, 0.59]
Intergenerational Congruence (Primary Outcome 3)				
Child self‐report	2.22 [−3.58, 8.01]	3.54 [−2.26, 9.35]	5.25 [−4.66, 15.15]	8.62 [−1.29, 18.52]
Caregiver report on child	0.16 [−0.32, 0.64]	0.00 [−0.50, 0.50]	0.04 [−0.27, 0.36]	−0.18 [−0.61, 0.24]
Parental Involvement (Primary Outcome 4.1)				
Child self‐report	−0.13 [−0.64, 0.37]	0.30 [−0.22, 0.82]	0.06 [−0.57, 0.69]	−0.09 [−0.77, 0.59]
Caregiver report on child	−0.04 [−0.46, 0.37]	0.21 [−0.23, 0.65]	0.01 [−0.48, 0.50]	−0.21 [−0.73, 0.30]
Positive Parenting (Primary Outcome 4.2)				
Child self‐report	−0.27 [−0.85, 0.32]	0.20 [−0.43, 0.83]	−0.26 [−0.89, 0.37]	−0.09 [−0.78, 0.60]
Caregiver report on child	−0.20 [−0.68, 0.27]	0.12 [−0.37, 0.60]	0.30 [−0.07, 0.66]	0.19 [−0.19, 0.57]
Poor Parental Monitoring/Supervision (Primary Outcome 4.3)				
Child self‐report	−0.23 [−0.78, 0.32]	−0.05 [−0.66, 0.57]	0.31 [−0.26, 0.87]	0.30 [−0.37, 0.97]
Caregiver report on child	−0.49 [−0.96, −0.02]	−0.10 [−0.61, 0.41]	Not converged	Not converged
Inconsistent Discipline (Primary Outcome 4.4)				
Child self‐report	0.31 [−0.24, 0.86]	−0.07 [−0.65, 0.51]	0.24 [−0.36, 0.84]	−0.22 [−0.92, 0.48]
Caregiver report on child	0.08 [−0.45, 0.61]	−0.32 [−0.94, 0.30]	0.05 [−0.43, 0.52]	−0.08 [−0.63, 0.47]
Corporal Punishment (Primary Outcome 4.5)				
Child self‐report^a^	1.20 [0.16, 8.82]	3.82 [0.42, 35.03]	Not converged	Not converged
Caregiver report on child^a^	0.67 [0.03, 14.86]	0.49 [0.02–15.87]	0.72 [0.10, 5.27]	1.89 [0.20, 18.15]
*Child Outcomes*				
Externalizing Behavior (Secondary Outcome 1)				
Child self‐report	−0.07 [−0.24, 0.10]	−0.00 [−0.18, 0.18]	0.14 [−0.13, 0.41]	0.19 [−0.10, 0.47]
Caregiver report on child	0.01 [−0.12, 0.14]	0.06 [−0.09, 0.20]	−0.32 [−0.90, 0.26]	0.07 [−0.48, 0.62]
Depression (CES‐DC) (Secondary Outcome 2)				
Child self‐report	0.11 [−0.08, 0.31]	−0.09 [−0.29, 0.11]	−0.18 [−0.58, 0.22]	−0.03 [−0.53, 0.48]
Caregiver report on child	0.01 [−0.09, 0.11]	−0.01 [−0.11, 0.10]	0.23 [−0.26, 0.71]	−0.06 [−0.51, 0.39]

*Note*: Coefficients are the difference in marginal means estimates for FSI‐R group at each timepoint.

Abbreviation: CI, confidence interval.

^a^ Mixed logistic estimation modeling was applied due to the highly skewed distribution of the outcome.

### Implementation outcomes

#### Fidelity and competence monitoring

Each interventionists received, on average, 3.93 (SD = 3.3) one‐on‐one fidelity monitoring feedback sessions out of 10 total sessions (Bhutanese monitoring sessions: mean = 4.75, SD = 0.65; Somali Bantu mean = 2.73, SD = 0.65) as well as weekly super‐supervision with the full cross‐site group and the Boston College researchers. Competence and fidelity scores were graded higher among Bhutanese than Somali Bantu interventionists overall (see Table 4). Assessments of competence ranged from 3.13 (SD = 0.80) at Module 1 to 3.69 (SD = 0.54) at Module 10 among Bhutanese group, and 2.96 (SD = 0.26) to 2.53 (SD = 0.46) among Somali Bantu group. Fidelity scores ranged from 2.84 (SD = 0.83) at Module 1 to 3.31 (SD = 0.92) at Module 10 among Bhutanese, and 1.98 (SD = 0.76) at Module 1 to 0.93 (SD = 0.46) at Module 10 among Somali Bantu. Overall, competence and fidelity were rated higher among Bhutanese interventionists than Somali Bantu interventionists (Table 4).

**Table 4:** Competence and fidelity scores reported by fidelity monitors by community.

Items	Bhutanese			Somali Bantu		
	*N*	Mean (SD)	Min/Max	*N*	Mean (SD)	Min/Max
Competence						
Module 1	21	3.13 (0.80)	0.2/4.0	9	2.96 (0.26)	2.4/3.4
Module 2	18	3.24 (0.51)	2.6/4.0	7	2.8 (0.28)	2.2/3.0
Module 3	17	3.39 (0.50)	2.4/4.0	7	2.57 (0.51)	1.8/3.0
Module 4	17	3.20 (0.35)	2.8/4.0	5	2.88 (0.18)	2.6/3.0
Module 5	17	3.29 (0.62)	2.0/4.0	3	2.53 (0.81)	1.6/3.0
Module 6	13	3.51 (0.42)	2.8/4.0	5	2.96 (0.09)	2.8/3.0
Module 7	12	3.63 (0.37)	3.0/4.0	6	2.93 (0.37)	2.2/3.2
Module 8	9	3.62 (0.48)	3.0/4.0	5	2.56 (0.52)	2.0/3.0
Module 9	7	3.63 (0.45)	3.0/4.0	2	2.6 (0.57)	2.2/3.0
Module 10	7	3.69 (0.54)	2.8/4.0	3	2.53 (0.46)	2.0/2.8
Fidelity						
Module 1	21	2.84 (0.83)	0.0/3.8	9	1.98 (0.76)	1.0/3.0
Module 2	18	3.17 (0.54)	2.4/4.4	7	1.54 (0.82)	0.6/2.6
Module 3	16	3.1 (0.37)	2.5/3.9	7	1.46 (0.62)	0.5/2.1
Module 4	16	2.87 (0.51)	1.9/3.8	5	2.05 (0.27)	1.6/2.4
Module 5	17	3.12 (0.56)	2.2/4.0	3	1.96 (0.84)	1.0/2.6
Module 6	12	3.25 (0.53)	2.4/4.0	5	2.43 (0.69)	1.4/3.3
Module 7	12	2.79 (0.77)	1.3/4.0	6	1.39 (0.56)	0.7/2.0
Module 8	9	2.89 (0.76)	1.3/3.7	5	0.93 (0.64)	0.0/1.7
Module 9	7	2.86 (0.75)	1.8/3.8	2	1.00 (0.71)	0.5/1.5
Module 10	7	3.31 (0.92)	1.8/4.0	3	0.93 (0.46)	0.4/1.2

#### SOFTA scale

The SOFTA subscale scores (i.e., engagement, emotional connection, safety, and purpose) are reported in Table 5. There was no difference in all the subdomain scores by type of agency nonspecialist delivery (existing CHWs or peers with lived experience hired and trained for the trial). When it came to SOFTA outcomes of emotional connection, scores were significantly higher for in‐person format (*t*(177) = 2.52, *p* = .01) among Somali Bantu families. No other differences were observed in subdomain scores across communities.

**Table 5:** SOFTA scores by community and delivery format: In‐person versus virtual, reported by interventionists.

Subscale	Bhutanese							Somali Bantu						
	In‐person			*Virtual*			Difference in‐person vs. virtual	In‐person			Virtual			Difference in‐person vs. virtual
	*N*	M (SD)	Min/Max	*N*	M (SD)	Min/Max	*t*(*df*)*	*N*	M (SD)	Min/Max	*N*	M (SD)	Min/Max	*t*(*df*)
Engagement	117	14.15 (1.68)	10/17	61	14.03 (1.99)	10/18	0.43 (176)	109	16.55 (2.92)	9/20	69	17.06 (2.58)	12/20	−1.16 (176)
Emotional Connection	117	13.20 (1.49)	9/17	61	13.13 (2.02)	10/18	0.27 (176)	110	17.4 (2.44)	10/20	69	16.41 (2.76)	10/20	2.52 (177)*
Safety	117	15.33 (2.15)	10/19	61	15.10 (2.33)	9/19	0.67 (176)	110	17.29 (2.56)	11/20	69	17.71 (2.24)	12/20	−1.18 (177)
Purpose	117	14.09 (1.92)	10/18	61	14.30 (2.07)	11/19	−0.92 (176)	110	16.32 (2.92)	9/20	67	16.41 (2.80)	9/20	−0.20 (175)

* *p* < .05.

## DISCUSSION

This study details the findings and process of a hybrid Type II implementation effectiveness trial of the FSI‐R carried out in two different refugee communities with different delivery models during the global COVID‐19 epidemic and a time of intense anti‐immigrant/refugee rhetoric and policies. At the family level, the present trial demonstrated that parental monitoring among Bhutanese families improved significantly in the intervention group compared to the Bhutanese CAU group. These results were not replicated among Somali Bantu families. In terms of implementation outcomes, routine supervision and quality improvement was seen to contribute to increased fidelity and competence among Bhutanese home‐visitors over time. In addition, a statistically significant association between in‐person delivery and sense of emotional connection among Somali Bantu families receiving in‐person delivery compared to online delivery. These results were not replicated among Bhutanese FSI‐R participants.

Despite significant external stressors including increased mental health challenges during COVID‐19 lockdowns and changes in refugee and immigrant policies, most outcomes were observed (without statistical significance) to trend in a positive direction in accordance with the intervention theory of change (Figure 2). Refugee groups have been hypothesized to be among some of the populations most severely affected by the consequences of COVID‐19 (Dubey et al., 2020). In this manner, stressors due to co‐occurring COVID‐19 and anti‐immigrant/refugee rhetoric in the United States at the time of this trial may have compounded mental health difficulties (Awaad et al., 2020; Chaudry et al., 2010; Ford‐Paz et al., 2020) making it difficult to determine intervention effects given the timing of this trial.

Also, due to the ongoing COVID‐19 pandemic underway during the time of this trial, parents may have had limited involvement in the community and schools and restricted their children's participation outside of home (Chávez et al., 2012; Sieffien et al., 2023; Singer et al., 2023. In addition, for many refugee families, suddenly having to support full‐time remote learning during lockdown was a considerable struggle (Heymann & Shindo, 2020), especially for non‐literate refugee parents and those unfamiliar with remote technology and online learning. This may have led to disruptions and tension in parent‐child relationships among refugee families (Ford‐Paz et al., 2020).

Other obstacles that hindered program implementation and data collection were similar to other studies conducted during the COVID‐19 pandemic including participant recruitment and retention (Akhtar et al., 2021; Bernardi et al., 2021; Celik et al., 2022; Miller et al., 2023; Saad et al., 2022), resulting in data loss. Despite utilizing imputation techniques to manage threats to address the smaller‐than‐anticipated sample, our data remains underpowered to draw definitive conclusions about the effectiveness outcomes included in this trial. Given the modest sample size and clustering within communities, the limitation is consistent with early‐stage Hybrid Type II trials, where the primary aim is to assess feasibility and preliminary effectiveness rather than achieve definitive statistical significance (Lévesque et al., 2020; Morrison et al., 2023). The failure to detect statistical significance in several of the outcomes in this trial cannot be taken to indicate that the intervention was not effective among Bhutanese and Somali Bantu refugee groups, as the impact of the global COVID‐19 pandemic led to massive sample size loss which may have attenuated our ability to detect statistically significant differences between the study groups.

Despite limitations, several insights can still be drawn from the Hybrid Type II implementation‐effectiveness design. First, our hybrid trial carried out in a context of social upheaval underscores the need for in‐depth understanding of the implementation ecology and the importance of implementation science framing (i.e., EPIS) to map out implementation barriers and facilitators at the organizational, community, and family levels. The EPIS framework allowed us to understand how relationships between the internal and external implementation context influenced one another. This analysis and the use of PDSA cycles to address implementation barriers helped the team to complete the hybrid trial despite significant challenges and helped to illuminate factors that can influence more successful program implementation in the future. Some of the key facilitators to successful implementation included the use of providers from the same cultural and linguistic background as the beneficiaries and focused sessions to carry out PDSA quality improvement cycles.

Our evaluation highlighted the significance of community collaboration in quickly adapting to addressing the needs of refugee groups when faced with challenges in the external environment. CBPR methods helped the research team to effectively problem solve major obstacles to implementation, including dramatic changes in refugee policies and services, and lockdowns due to the global COVID‐19 pandemic which necessitated moving both intervention and data collection to remote formats.

Moreover, the implementation of community support initiatives led by CBOs was crucial, to support families who faced significant challenges in adapting to new environments while also navigating the closure and reopening of schools in the aftermath of the COVID‐19 pandemic. To establish confidence and secure the commitment of the participants, it was critical to advocate for support and services beyond the immediate focus of the FSI‐R to uphold the intention of the CBPR partnership. For example, many of the adult caregivers had limited proficiency in the English language, impeding their ability to actively engage in their child's academic endeavors and participate effectively in available support services. Advocating for the resourcing of school interpreters to address language obstacles was an important part of supporting better overall navigation of refugee caregivers in their schools including during the COVID‐19 pandemic (Thakur et al., 2020).

Overall, EBI implementation research grounded within a CBPR methodology can help deepen knowledge of culture, language, and community needs to better refine models for ensuring reach of EBIs and quality improvement in delivery over time. Being involved in community partnerships creates a supportive atmosphere for the implementation and sustainment of evidence‐based family strengthening initiatives in communities that may not receive attention otherwise (Albahsahli et al., 2023). A strong community partnership contributed to the hybrid type II implementation components, such as examining different types of staffing configurations (existing CHWs vs. nonspecialists hired from the community) and delivery formats (in‐person vs. remote), as shown by improving scores of fidelity, competence, and SOFTA ratings over time. Our findings demonstrate that even during a crisis, implementation of FSI‐R was feasible and acceptable and had the potential to be effectively implemented by a diverse group of workers when delivered under consistent supervision and monitoring both in virtual and in‐person settings.

## CONCLUSION

We used a Hybrid Type II Study guided by the EPIS model and CBPR approaches to study the implementation and effectiveness of FSI‐R during the global COVID‐19 pandemic and political upheaval affecting migrants and refugees. The Boston College Institutional Review Board (Protocol #18.251.01) granted permission for the study, and informed consent has been appropriately obtained from all participants. Our effectiveness results indicate enhancements in parental monitoring among Bhutanese families and greater preference for in person as opposed to remote delivery among Somali Bantu refugees. Well‐trained and supervised refugee home visitors were able to improve both fidelity and competence with time under both delivery strategies (i.e., facilitation by CHWs already embedded in an agency compared to facilitators hired from the community for the purposes of this study). Our process evaluation, guided by the EPIS model examining both the external context of implementation ecosystem and the internal context of the CBOs documented how multi‐level, culturally sensitive, and access‐oriented policies and programs across sectors helped buffer some deleterious effects of the pandemic and political environment (Browne et al., 2021; Ford‐Paz et al., 2020).

The CBPR process and attention to the implementation ecosystem provided a model for adapting the intervention to cultures and ever‐changing contexts and a method for making delivery as nimble as possible. Our research indicates that the FSI‐R had good acceptability among refugee families and was feasible despite the influence of the global COVID‐19 pandemic and negative social attitudes, policies, and political rhetoric about refugees and immigrants in the United States. Our CBPR partnership will continue to play a crucial role in facilitating more innovation and expansion of the FSI‐R with greater reach and sustained quality. Future research is needed with adequate sample size to draw more definitive conclusions about the ongoing impact of the intervention as it is scaled to a greater number of families across a range of cultures.

## AUTHOR CONTRIBUTIONS

Euijin Jung led the manuscript writing, analysis and review process. Candace Black contributed substantially by writing and reviewing the manuscripts. Matias Palcencio‐Castro provided important insights for the analysis and writing of the results. Lila Chamlagai contributed substantially by writing and reviewing the manuscripts. Rilwan Osman contributed substantially by reviewing and provided insights to improve contextual understanding of the manuscripts. Morgan Hoffman contributed substantially by reviewing the manuscripts. William Beardslee contributed substantially by writing and reviewing the manuscripts. Theresa Betancourt contributed substantially by writing and reviewing the manuscripts.

## CONFLICT OF INTEREST STATEMENT

The authors declare no conflicts of interest.

## ETHICS STATEMENT

The Boston College Institutional Review Board (Protocol #18.251.01) granted permission for the study.

## Supporting information

Supporting information.

Supporting information.

## Data Availability

The data that support the findings of this study are available upon reasonable request. The data that support the findings of this study are available from the corresponding author upon reasonable request.

## Footnotes

1 In a Type I Hybrid study, the primary aim is to test the clinical effectiveness of an intervention while also gathering some preliminary data on implementation. In a Type III Hybrid study, the primary focus is on testing implementation strategies, while collecting some secondary data on intervention outcomes. A Type II Hybrid study gives equal weight to both aims, meaning that it tests whether the intervention works (effectiveness) and examines how it is implemented (fidelity, feasibility, barriers, facilitators) in real‐world settings at the same time.
