# Supplementary material for: Delivering a family‐based child mental health promotion program among two resettled refugee communities during the COVID‐19 pandemic: Lessons learned in a hybrid type II implementation‐effectiveness randomized controlled trial
Source: Am J Community Psychol. 2025 Oct 5;77(1-2):231–47. doi: 10.1002/ajcp.70021 (PMC13007756; doi:10.1002/ajcp.70021)
Supplement: Supplementary file 1 — Supporting information. [file AJCP-77-231-s001.docx]

# **Appendix I: FSI-R Modules**

| **Modules** | **Theme(s)** | **Description of Contents** | **Aims** |
| --- | --- | --- | --- |
| **1 -2** | **Introduction** | - Introduction to the intervention’s goals & structure - Creation & discussion of a “Family Narrative” - Identification of family strengths to build upon - Identification of family goals | - To prepare caregivers for intervention - To motivate family’s participation and investment - To begin to establish a trusting relationship with caregivers - To understand family’s unique circumstances |
| **3** | **Children and Family Relationships** | - Creation and discussion of a “Family Narrative” from a child’s point of view to guide the intervention. - Identification of family strengths and challenges - Build communication skills in preparation for family meeting | - To begin to establish a trusting relationship with children - To understand family’s unique circumstances from a child’s point of view |
| **4** | **Responsive parenting and caregiving** | - Identify ways to engage and respond to children, build and maintain positive parent-child relationships, and encourage desirable behavior. - Identify and model ways to discipline children and alternatives to harsh punishment - Explain importance of adults’ active involvement and communication with their children | - To understand family dynamics and routines - To introduce specific strategies to manage stress and reduce harsh punishments |
| **5** | **Engagement with the US education system** | - Importance of engagement in the US education system and discussing school with children - Update of family narrative with focus on school experiences - Coaching on specific, age-appropriate structured activities families can incorporate into their daily routines to talk to their children about school | - To educate caregivers on the education system in the US - To empower caregivers to engage with schools and their children’s educational experience |
| **6** | **Supplemental Module: Promoting Health, Wellbeing and Safety** | - Discussion and demonstration of stress management - Guide for healthy eating and engaging in physical activity - Discussion of household hygiene - Effective strategies for prevention and wellness - Identification of the health risks of excessive alcohol consumption - Staying safe in the community and at home | - To help caregivers learn about good physical and mental health - To identify strategies that promote health, wellbeing and safety |
| **7-8** | **Communicating with Children and Caregivers** | - Building communication skills - Identifying ways to respond well to hard questions - Strategies for building stronger families - Preparing for Family Meeting | - To prepare caregivers and children for the Family Meeting - To build skills related for improved child-parent communication |
| **9** | **Uniting the Family** | - Family Meeting - Create shared understanding of resettlement and focus on family strengths - Recognize each family member’s experience and views | - To promote positive communication between children and caregivers |
| **10** | **Bringing It All Together** | - Review the content of previous modules - Create a plan with the family for how they will apply what they have learned going forward - Discuss ways to involve all caregivers and family members in these strategies, activities and routines | - To empower the family to practice and implement new strategies and skills learned throughout the intervention |

# **Appendix II.** **Main Outcomes at Each Timepoint by Intervention Status**

|  | **Intervention** | | | | | | | **Care-As-Usual** | | | | | | | | |
| --- | --- | --- | --- | --- | --- | --- | --- | --- | --- | --- | --- | --- | --- | --- | --- | --- |
|  | Baseline | | Midline | | | Endline | | Baseline | | | | Midline | | Endline | | |
| **Outcomes** | Mean | SD | Mean | SD | Mean | | SD | | Mean | SD | Mean | | SD | Mean | SD | |
| **Family Outcome at Baseline** | | | | | | | | | | | | | | | | |
| **Family conflict** |  |  |  |  |  | |  | |  |  |  | |  |  |  | |
| Child self-report | 0.69 | 1.21 | 0.60 | 1.12 | 0.03 | | 0.11 | | 0.78 | 1.26 | 0.56 | | 1.03 | 0.16 | 0.37 | |
| Caregiver Report | 0.27 | 0.52 | 0.14 | 0.35 | 0.36 | | 1.10 | | 0.15 | 0.39 | 0.13 | | 0.42 | 0.36 | 0.88 | |
| **Parental Efforts for Communication** | | | | | | | | | | | | | | | | |
| Child self-report | 3.67 | 0.86 | 3.90 | 0.84 | 4.44 | | 0.58 | | 3.61 | 0.75 | 3.68 | | 0.83 | 3.75 | 0.52 | |
| **Parent-Child Open Communication** | | | | | | | | | | | | | | | |  |
| Child self-report | 3.44 | 1.16 | 3.63 | 1.05 | 4.00 | | 0.83 | | 3.26 | 1.13 | 3.36 | | 1.01 | 3.40 | 0.91 | |
| **Intergenerational congruence** | | | | | | | | | | | | | | | | |
| Child self-report | 5.33 | 6.07 | 4.14 | 0.76 | 4.77 | | 0.34 | | 8.18 | 13.78 | 5.62 | | 8.85 | 4.07 | 0.92 | |
| Caregiver Report | 3.59 | 0.86 | 3.82 | 0.78 | 3.98 | | 0.80 | | 3.30 | 0.96 | 3.45 | | 0.83 | 3.45 | 0.88 | |
| **Parental Involvement** |  |  |  |  |  | |  | |  |  |  | |  |  |  | |
| Child self-report | 3.30 | 0.97 | 3.47 | 0.89 | 3.93 | | 0.66 | | 3.00 | 0.89 | 3.34 | | 0.84 | 3.24 | 0.45 | |
| Caregiver Report | 3.64 | 0.69 | 3.71 | 0.82 | 4.15 | | 0.62 | | 3.43 | 0.69 | 3.65 | | 0.72 | 3.57 | 0.51 | |
| **Positive Parenting** |  |  |  |  |  | |  | |  |  |  | |  |  |  | |
| Child self-report | 3.72 | 0.97 | 3.76 | 0.93 | 4.46 | | 0.65 | | 3.46 | 0.96 | 3.71 | | 0.92 | 3.61 | 0.56 | |
| Caregiver Report | 4.19 | 0.69 | 4.14 | 0.71 | 4.54 | | 0.62 | | 4.16 | 0.76 | 4.06 | | 0.72 | 3.93 | 0.66 | |
| **Poor Parental Monitoring/Supervision** | | | | | | | | | | | | | | | | |
| Child self-report | 1.71 | 0.61 | 1.69 | 0.56 | 1.66 | | 0.59 | | 1.95 | 0.66 | 1.98 | | 0.74 | 1.84 | 0.77 | |
| Caregiver Report | 1.63 | 0.64 | 1.60 | 0.51 | 1.85 | | 0.71 | | 1.60 | 0.61 | 1.75 | | 0.69 | 1.65 | 0.64 | |
| **Inconsistent Discipline** | | | | | | | | | | | | | | | | |
| Child self-report | 1.88 | 0.65 | 2.10 | 0.75 | 1.70 | | 0.45 | | 1.76 | 0.62 | 1.90 | | 0.87 | 1.79 | 0.74 | |
| Caregiver Report | 1.87 | 0.65 | 1.85 | 0.60 | 1.77 | | 0.86 | | 1.72 | 0.48 | 1.94 | | 0.74 | 1.88 | 0.66 | |
| **Corporal Punishment** |  |  |  |  |  | |  | |  |  |  | |  |  |  | |
| Child self-report | 1.11 | 0.21 | 1.19 | 0.48 | 1.16 | | 0.26 | | 1.33 | 0.61 | 1.40 | | 0.85 | 1.11 | 0.22 | |
| Caregiver Report | 1.13 | 0.39 | 1.13 | 0.29 | 1.13 | | 0.36 | | 1.11 | 0.31 | 1.36 | | 0.69 | 1.11 | 0.26 | |
| **Child Outcome at Baseline** | | | | | | | | | | | | | | | | |
| **Externalizing Behavior** | | | | | | | | | | | | | | | | |
| Child self-report | 1.23 | 0.24 | 1.23 | 0.22 | 1.24 | | 0.23 | | 1.29 | 0.31 | 1.20 | | 0.24 | 1.11 | 0.15 | |
| Caregiver Report | 1.15 | 0.23 | 1.16 | 0.19 | 1.25 | | 0.23 | | 1.16 | 0.23 | 1.22 | | 0.78 | 1.13 | 0.19 | |
| **Depression (CES-DC)** | | | | | | | | | | | | | | | | |
| Child self-report | 0.21 | 0.35 | 0.21 | 0.28 | 0.18 | | 0.33 | | 0.33 | 0.50 | 0.24 | | 0.41 | 0.21 | 0.29 | |
| Caregiver Report | 0.60 | 0.49 | 0.67 | 0.48 | 0.53 | | 0.51 | | 0.70 | 0.46 | 0.64 | | 0.49 | 0.71 | 0.46 | |

# **Appendix III.** EPIS Framework Application for FSI-R

| **Inner Context** | **Outer Context** | **Bridging Factors** | **Innovation Factors** |
| --- | --- | --- | --- |
| **Individual Characteristics**  · Staff have other personal challenges (transportation)  · Staff have other commitment (maternity leaves, families events, holidays to Africa or Nepal)  **Organizational Characteristics**  · Not enough resources for full-time placement  · In need of financial specialist  **Organizational Staffing Processes**  · Hired interns from local colleges → supervision needed (Somali Bantu)  · Frequent staff turnover→ staff trained as both interventionists and RAs  **Quality and Fidelity Monitoring/Support**  · Field staff needs tech training  · Weekly check-in with fidelity monitors  · Fidelity monitoring is very time-consuming (streamlining needed, e.g., picking the hardest modules)  · **After Covid:** sessions over the phone was successful  · **After Covid:** for data collection, tablets and hotspots were provided  · **After Covid:** streamlining the data collection process from the big family (e.g., collecting only one adult and one child) | **Client Characteristics**  · Hard to provide services during Ramadan (Somali Bantu)  · Complex communities and  family configuration  · Scheduling issue (e.g., hard to set up a time to meet due to long sessions)  · High illiteracy rates→ parents showing difficulty supporting children learning  · For exit interviews, families are reluctant as they do not know the exit interviewers (Somali Bantu)  · Lots of stigma, intergenerational divide were reported (Bhutanese)  · **After Covid**: families are overwhelmed by school opening  · **After Covid**: Most families do not have internet at home  · **After Covid:** Facebook live on COVID awareness was very successful (Bhutanese)  · **After Covid:** Families mental health worsens due to Covid (e.g., triggering PTSD from past experiences of oppression and being victims of racist violence, feelings of anxiety, loss of loved ones; loss of livelihoods)  **Service Environment/Policies**  · Local resource building (e.g., school interpreter to overcome language barriers and communication, adding domestic violence resources, adding grants/scholarship opportunities to the resource guide to students  · Alcoholism is growing concern  · Fire in the community (Somali Bantu)  · Racial disparity on covid cases in ME (Somali Bantu)  · **After Covid:** Harsh political climate toward refugees  **Funding/Contracting**  · Continuous grant-seeking | **Community Academic Partnership**  · Separate Youth and Adult CAB  · Youth group into two separate age range suggested by clinical director (Somali Bantu)  · Reached out to Islamic Society of Boston for Alcoholism support group  · **After Covid:** Facebook live was very successful on Covid Awareness (Bhutanese)  · **After Covid:** Public health and parenting videos during Covid were uploaded and distributed through social media (Bhutanese)  · **After Covid**: CAB in zoom | **Innovation/EBP developers**  · Family size is big→ harder for data collection (Somali Bantu)  · Actively refining manuals based on the feedback from CAB (simpler language, more verbatim on what should be shared, more commentary for interventionists)  · **After Covid:** over-the-phone delivery prepared  · **After Covid:** Developed COVID measures  **Innovation/EBT Fit System**  · Automatic voice recording system set up for recording the session  · Digitizing consent forms in the REDCap  · Audio-recording via phone feature was prepared  · **After Covid:** over the phone or zoom data collection (Script was prepared and approved; not to exceed 20 minutes at a time  · **After Covid:** Tip-sheets for using technology was prepared  **Innovation/ EBT Characteristics**  · Workshop on alcohol related topic was held  · Training staff on addressing sensitive topics (e.g., Alcohol).  · Resources for teens were prepared  · Resources on having conversations (with kids) about race/racisms were developed |
