# Supplementary material for: Delivering a family‐based child mental health promotion program among two resettled refugee communities during the COVID‐19 pandemic: Lessons learned in a hybrid type II implementation‐effectiveness randomized controlled trial
Source: Am J Community Psychol. 2025 Oct 5;77(1-2):231–47. doi: 10.1002/ajcp.70021 (PMC13007756; doi:10.1002/ajcp.70021)
Supplement: Supplementary file 2 — Supporting information. [file AJCP-77-231-s002.docx]

**The Consort 2010 Checklist** (<https://www.ncbi.nlm.nih.gov/pmc/articles/PMC6398298/#:~:text=The%20CONSORT%20statement%20is%20made,%2C%20publishing%2C%20research%2C%20research%20design>)

| **Section/topic** | **Item number** | **Checklist item** | **Status** |
| --- | --- | --- | --- |
| **Title and abstract** | 1a | Identification as a randomized trial in the title | Done (on title and throughout the manuscript) |
|  | 1b | Structured summary of trial design, methods, results, and conclusions | Done |
| **Introduction** |  |  |  |
| Background and objectives | 2a | Scientific background and explanation of the rationale | Done (p1-2) |
|  | 2b | Specific objectives or hypotheses | Done (p.8) |
| **Methods** |  |  |  |
| Trial design | 3a | Description of trial design (such as parallel, factorial) including allocation ratio | Done (p.10) |
|  | 3b | Important changes to methods after trial commencement (such as eligibility criteria), with reasons | Done (p.10 and throughout the manuscript) |
| Participants | 4a | Eligibility criteria for participants | Done (p.10) |
|  | 4b | Settings and locations where the data were collected | Done (p.2-3,10,12) |
| Interventions | 5 | The interventions for each group with sufficient details to allow replication, including how and when they were actually administered | Done (p.10-12) |
| Outcomes | 6a | Completely defined pre-specified primary and secondary outcome measures, including how and when they were assessed | Done (p.13) |
|  | 6b | Any changes to trial outcomes after the trial commenced, with reasons | NA |
| Sample size | 7a | How sample size was determined? | Done (p.12) |
|  | 7b | When applicable, explanation of any interim analyses and stopping guidelines | NA |
| **Randomization** |  |  |  |
| Sequence generation | 8a | The method used to generate the random allocation sequence | Done (p.12) |
|  | 8b | Type of randomization; details of any restriction (such as blocking and block size) | Done (p.12) |
| **Allocation concealment mechanism** | 9 | The mechanism used to implement the random allocation sequence (such as sequentially numbered containers), describing any steps taken to conceal the sequence until interventions were assigned | Done (p.12) |
| **Implementation** | 10 | Who generated the random allocation sequence, who enrolled participants, and who assigned participants to interventions | Done (p.12) |
| **Blinding** | 11a | If done, who was blinded after assignment to interventions (e.g., participants, care providers, those assessing outcomes) and how | Done (p.10) |
|  | 11b | If relevant, description of the similarity of interventions | NA |
| **Statistical methods** | 12a | Statistical methods used to compare groups for primary and secondary outcomes | Done (p.13) |
|  | 12b | Methods for additional analyses, such as subgroup analyses and adjusted analyses | Done (p.13) |
| **Results** |  |  |  |
| Participant flow (a diagram is strongly recommended) | 13a | For each group, the numbers of participants who were randomly assigned received intended treatment and were analyzed for the primary outcome | Figure 3 |
|  | 13b | For each group, losses and exclusions after randomization, together with reasons | Done (P.12) |
| Recruitment | 14a | Dates defining the periods of recruitment and follow-up | Done (p.10) |
|  | 14b | Why the trial ended or was stopped | Done (p.10) |
| Baseline data | 15 | A table showing the baseline demographic and clinical characteristics for each group | Table 1, P13-14 |
| Numbers analyzed | 16 | For each group, number of participants (denominator) included in each analysis and whether the analysis was by original assigned groups | Table 1,2,3, Figure 3 |
| Outcomes and estimation | 17a | For each primary and secondary outcome, results for each group, and the estimated effect size and its precision (such as 95% confidence interval) | Done (Table3) |
|  | 17b | For binary outcomes, presentation of both absolute and relative effect sizes is recommended | Done |
| Ancillary analyses | 18 | Results of any other analyses performed, including subgroup analyses and adjusted analyses, distinguishing pre-specified from exploratory | NA |
| Harms | 19 | All important harms or unintended effects in each group (for specific guidance see CONSORT for harms) | Done |
| **Discussion** |  |  |  |
| Limitations | 20 | Trial limitations, addressing sources of potential bias, imprecision, and, if relevant, the multiplicity of analyses | Done (p.17-21) |
| Generalizability | 21 | Generalizability (external validity, applicability) of the trial findings | Done (p.17-21) |
| Interpretation | 22 | Interpretation consistent with results, balancing benefits and harms, and considering other relevant evidence | Done (p.17-21) |
| **Other information** |  |  |  |
| Registration | 23 | Registration number and name of trial registry | Done (p.8) |
| Protocol | 24 | Where the full trial protocol can be accessed, if available | Done (title page) |
| Funding | 25 | Sources of funding and other support (such as the supply of drugs), the role of funders | Done (title page, acknowledgement) |
